# Enhancing cross-context generalization in drug perturbation prediction with a multimodal conditional diffusion framework

**DOI:** 10.1093/bioinformatics/btag482

**Published:** 2026-06-30

**Authors:** Yanjie Ma, Kang Du, Yan Li, Pengyong Li, Liang Yu

**Affiliations:** School of Computer Science and Technology, Xidian University, Xi’an, 710126, Shaanxi, China; School of Computer Science and Technology, Xidian University, Xi’an, 710126, Shaanxi, China; School of Computer Science and Technology, Xidian University, Xi’an, 710126, Shaanxi, China; School of Management, Xi’an Polytechnic University, Xi’an, 710000, Shaanxi, China; School of Computer Science and Technology, Xidian University, Xi’an, 710126, Shaanxi, China; School of Computer Science and Technology, Xidian University, Xi’an, 710126, Shaanxi, China

## Abstract

**Motivation:**

Predicting drug-induced transcriptional perturbations is critical for precision medicine, yet existing models fail to capture multimodal biological context, limiting generalization across unseen drugs and cell lines.

**Results:**

We present PertDiff, a conditional diffusion framework that integrates control gene expression, LLM-derived cell semantics, and pretrained molecular graph representations to predict transcriptome-wide perturbations. PertDiff outperforms state-of-the-art baselines in prediction accuracy and generalizes robustly across drugs and cell lines. It further demonstrates translational utility through accurate drug sensitivity prediction, therapeutic repurposing for pancreatic cancer, and concordance with real-world clinical treatment outcomes, establishing it as a biologically grounded transcriptomic modeling tool.

**Availability:**

The source code and data are available at https://github.com/Panda-myj/PertDiff and https://doi.org/10.5281/zenodo.18427848.

## 1 Introduction

Disease heterogeneity challenges the effectiveness of conventional treatments, making it essential to uncover how drugs reshape cellular states (Qi *et al.* 2024, Tong *et al.* 2024). However, conventional in vitro screening assays involve exhaustive experimentation over a vast number of drug-cell combinations, rendering them costly, time-consuming, and impractical for exploring the full combinatorial spaces (Drews 2000). Fortunately, the emergence of large-scale transcriptomic datasets has opened new avenues for computational modeling, offering a data-driven alternative to predict drug-induced cellular responses at scale (Basu *et al.* 2013, Yang *et al.* 2013, Pham *et al.* 2021, Wu *et al.* 2022, Zeng *et al.* 2022, Huang *et al.* 2023, 2025, Ren *et al.* 2024, Roohani *et al.* 2024).

The core computational task in this domain is transcriptional perturbation prediction, given a control gene expression profile and a specific drug, the model predicts the corresponding perturbed gene expression. Successfully solving this problem enables the in silico estimation of transcriptome-wide cellular responses, providing a powerful tool for identifying potential therapeutics.

Deep learning has recently emerged as a promising approach for this task (Li *et al.* 2021, Yang *et al.* 2022, Qin *et al.* 2024, Qi *et al.* 2025, Qiao *et al.* 2025), yet limitations persist in current methodologies. Most existing models rely on Variational Autoencoders (VAEs), such as TranSiGen and PRnet. While effective for dimensionality reduction, VAEs frequently suffer from posterior collapse and generate “blurred” predictions, struggling to capture the subtle, high-frequency variations that characterize biological perturbations (Lucas *et al.* 2019, Wang *et al.* 2021, Qi *et al.* 2024, Tong *et al.* 2024, Zhao *et al.* 2024). Although diffusion-based models such as CrossDiT and CatCrossDiT have recently demonstrated superior generative quality, they often overlook critical biological priors (Hu *et al.* 2026). Specifically, these models typically fail to leverage graph-based pretrained networks for molecular representation, and they define cell identity solely based on baseline gene expression. This formulation ignores the rich semantic context, such as tissue origin, disease type, which profoundly influences how a cell responds to pharmacological intervention (Li *et al.* 2023, Qiao *et al.* 2024, Liu *et al.* 2025).

To address these challenges, we propose PertDiff, a conditional diffusion framework designed to overcome the limitations of VAE-based approaches while explicitly integrating rich multimodal contexts (Ho *et al.* 2020, Zhang *et al.* 2023). PertDiff innovates by conditioning the generative process on three distinct information sources: (1) control gene expression, (2) molecular structural features extracted via a pretrained graph neural network (MolGNet) (Li *et al.* 2021), and (3) semantic cell descriptions derived from Large Language Model (LLM) (Reimers and Gurevych 2019, DeepSeek 2025, OpenAI 2025). By jointly modeling the interaction between chemical structure and cellular semantic context, PertDiff enhances the model’s ability to generalize to unseen drugs and cell lines, enabling robust and biologically coherent inference.

We systematically evaluated PertDiff through a rigorous multi-stage validation. First, we demonstrated its superior accuracy in transcriptomic prediction against state-of-the-art baselines. Subsequently, we validated its pharmacological relevance through diverse applications, including: (1) accurate drug sensitivity prediction; (2) therapeutic repurposing for pancreatic cancer and (3) validation against real-world clinical drug responses.

## 2 Methods

### 2.1 Data source and feature construction

Gene expression data were obtained from the CMAP LINCS 2020 dataset released by the Broad Institute, a large-scale resource of perturbational transcriptomic profiles (Lamb *et al.* 2006, Subramanian *et al.* 2017, Broad Institute 2020). We used the processed level-3 data provided by TranSiGen and selected samples with 10 μM drug concentration and 24 h treatment (Tong *et al.* 2024). Perturbed samples were paired with plate-matched controls to reduce batch effects, and replicates were aggregated using the MODZ algorithm [a robust z‑score based aggregation method that combines replicated L1000 measurements into a single consensus signature (Subramanian *et al.* 2017)]. After preprocessing, the dataset comprised 78 569 drug-cell combinations covering 164 cell lines and 8316 drugs, each with control and perturbed expression of 978 landmark genes. Gene expression values were linearly transformed to [−1, 1] to align with the zero‑mean Gaussian noise assumption in the diffusion forward process, following the common scaling practice in diffusion-based generative modeling.

Drug representations were obtained using the pretrained MolGNet model, which encodes molecular graphs derived from Simplified Molecular Input Line Entry System (SMILES) strings to produce graph-level embeddings capturing structural and chemical information (Li *et al.* 2021).

Cell line descriptions were generated and curated using large language models and manually verified. These final descriptions were encoded into 384-dimensional semantic embeddings using the all-MiniLM-L6-v2 Sentence-Transformers model (Reimers and Gurevych 2019). These embeddings were integrated with gene expression features in the model.

Additional details on dataset preprocessing and feature construction are provided in the Supplementary Materials.

### 2.2 PertDiff model architecture

**Figure 1:**
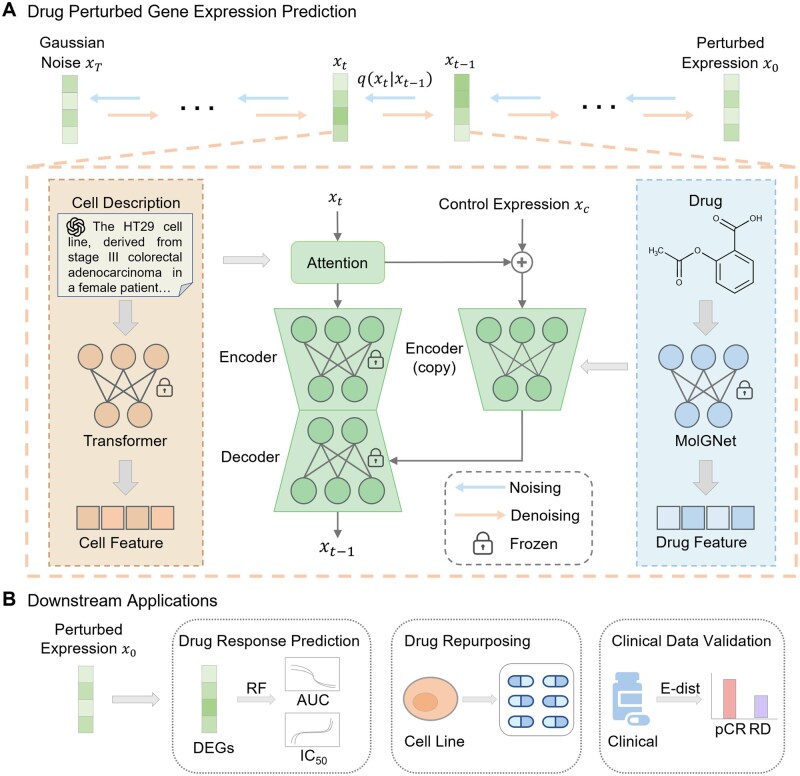
Overview and applications of the PertDiff model. (A) Illustration of the noising and denoising processes. In the forward noising process, the observed perturbed gene expression profile is corrupted by adding noise at randomly selected time steps. During the reverse denoising process, the model starts from Gaussian noise and progressively denoises it under the conditioning of control expression, drug, and cell description. At each step t, the model predicts ${\hat{x}}_{\theta }(x_{t},t)$ to estimate the mean and variance of the previous state distribution, resamples accordingly, and finally constructs the mean of the distribution at the final step (t=0), corresponding to the predicted perturbed gene expression. (B) Applications of the predicted perturbed gene expression, including drug response prediction, drug repurposing, and clinical data validation.

We propose PertDiff, as shown in Fig. 1A, a multimodal perturbation-prediction model built on the Denoising Diffusion Probabilistic Model (DDPM) framework and implemented with a conditioning design (Ho *et al.* 2020, Zhang *et al.* 2023). The model performs conditional denoising of gene-expression: (i) control expression and drug are fused through a conditional network that supplies modality-specific features to the denoiser, and (ii) cell descriptions are encoded into contextual embeddings and fused via a cross-attention mechanism that modulates the denoising layers (Vaswani *et al.* 2017). These three information streams, control expression, drug, and cell description are fused within the network so that the denoising process is explicitly guided to generate biologically plausible perturbed gene expression. The PertDiff architecture preserves the probabilistic sampling advantages of DDPMs while enabling interpretable conditioning from heterogeneous biological modalities.

#### 2.2.1 Learning to reverse perturbed expression

**Forward noising.** During forward noising process, we sample a diffusion timestep t from 1 to T uniformly and corrupt the observed perturbation gene expression $x_{0}$ with Gaussian noise to obtain $x_{t}$. We set T=1000 and adopt the cosine noise schedule (Nichol and Dhariwal 2021) to define $\alpha_{t}$. Following the DDPM formulation, the forward process is written as:


(1) q(xt|x0)=N(xt;α¯tx0,(1-α¯t)I)

which equivalently corresponds to sampling


(2) xt=α¯tx0+1-α¯tz, z∼N(0,I)

Here ${\bar{\alpha }}_{t}\in (0,1]$ denotes the cumulative scaling factor at timestep t (commonly defined as ${\bar{\alpha }}_{t}=\prod_{s=1}^{t}\alpha_{s}$ from a prescribed schedule $\left\{\alpha_{s}\right\}$), and T is the total number of diffusion steps. The schedule for ${\bar{\alpha }}_{t}$ is chosen so that as t→T the signal is progressively extinguished and $x_{T}$ approaches isotropic Gaussian noise. Equation (1) defines the conditional distribution used to train the denoising model to recover $x_{0}$ from noisy observations $x_{t}$.

**Reverse denoising.** In the reverse denoising process, the network is trained to predict the observed perturbed gene-expression from its noisy observation $x_{t}$.

Given a noised input $x_{t}$, the model first projects it through a multi-layer perceptron (MLP) to a 978-dimensional latent representation $x_{l}$, which is then integrated with the cell description feature via a multi-head cross-attention mechanism. In the multi-head cross-attention mechanism, the 384-dimensional cell description feature is embedded into a 128-dimensional and replicated 978 times to match the gene dimension. After adding a learnable positional encoding, the resulting cell description feature matrix serves as the query (Q) with shape (978 128). Meanwhile, the $x_{l}$ is reshaped to (978,1) and each element is then linearly mapped to form the key (K) and value (V) matrices, each of shape (978 128). The attention computation follows the standard scaled dot-product formulation:


(3) headi=Attention(Qi,Ki,Vi)=softmax(QiKiTdk)Vi, i=1,2,3,4

where four attention heads (h=4) are used, each with a subspace dimension $d_{k}=32$. The outputs from all heads are concatenated as


(4) MultiHead(Q,K,V)=Concact(head1,…,head4)

yielding an output of shape (978 128), which is then linearly projected back to (978,1) and subsequently flattened into a 978-dimensional feature $x_{ca}$, then passed to the subsequent conditional fusion modules.

The control expression $x_{c}$ and drug feature are fused through a conditional control network. Specifically, $x_{c}$ is processed by an MLP followed by a learnable zero-convolution layer (zero‑initialized fully connected layer), and its output is added element-wise to the $x_{ca}$, thus obtaining $x_{\mathrm{cca}}$. Simultaneously, the drug features are processed through an MLP and then concatenated with the time features, the combined feature is then remapped to the original dimensionality of the time embedding, thus obtaining $x_{dt}$, replacing the original time-feature in copy encoder network. Both $x_{\mathrm{cca}}$ and $x_{dt}$ are further processed through a copy but learnable conditional encoder followed by additional zero convolution layers, after which their features are fused with the corresponding decoder features of the decoder network via additive connections. This design ensures full conditional control across the denoising trajectory, enabling the model to generate biologically consistent perturbation outcomes guided by cell-, drug-, and time-dependent contexts. The final network output is the model’s estimate of the denoised gene expression, denoted as ${\hat{x}}_{\theta }(x_{t},t)$.

#### 2.2.2 Predicting perturbations via diffusion sampling

sampling begins at t=T by sampling an initial 978-dimensional feature $x_{T}$ from a standard gaussian:


(5) xT∼N(0,I)

At each reverse step t, the model performs a denoising process conditioned on the control expression, drug and cell description to produce a prediction of the cleaner expression ${\hat{x}}_{\theta }(x_{t},t)$. This predicted ${\hat{x}}_{\theta }(x_{t},t)$ is then used to form the posterior distribution $q(x_{t-1}|x_{t},\;{\hat{x}}_{\theta }(x_{t},t))$.

Following the DDPM posterior closed form, the posterior mean $\mu_{q}(x_{t},\;{\hat{x}}_{\theta }(x_{t},t))$ and variance ${\tilde{\beta }}_{q}$ at step t−1 are


(6) μq(xt,x^θ(xt,t))=α¯t−1βt1-α¯tx^θ(xt,t)+αt(1-α¯t−1)1-α¯txt

(7) β∼q=1-α¯t−11-α¯tβtI

$$\text{where}\;\beta_{t}=1-\alpha_{t}\text{.}$$

Sampling


(8) xt−1=μq(xt,x^θ(xt,t))+β∼qz, z∼N(0,I)

This sampling step is then iterated backward in time: the sampled $x_{t-1}$ becomes the input to the network at the next iteration, which yields ${\hat{x}}_{\theta }(x_{t-1},t-1)$ and a new posterior for $x_{t-2}$, and so on. At the final step (t=1), the posterior mean $\mu_{q}(x_{1},{\hat{x}}_{\theta }(x_{1},1))$ provides the point estimate of the predicted perturbed expression.

This conditioned sampling procedure ensures that the generated 978-dimensional gene expression is progressively denoised while remaining controlled by the control expression, drug and cell description, yielding biologically coherent predictions of the perturbed gene expression.

#### 2.2.3 Implementation details

The PertDiff model was trained in two stages: pretraining and fine-tuning. During pretraining, the encoder–decoder backbone was trained to reconstruct clean gene expression from noisy inputs using a mean squared error (MSE) objective. In the fine-tuning stage, conditional modules incorporating control expression, drug features, and cell description embeddings were introduced to guide the denoising process. The MSE is used for fine-tuning, comparing the predicted perturbed expression against the observed.

Model optimization employed a smaller learning rate during fine-tuning with a two-phase decay schedule consisting of linear decay followed by cosine annealing. Parameters were initialized using standard strategies, including pretrained weights for the backbone network and Xavier initialization for newly introduced layers.

Full implementation details, training procedures, and hyperparameters are provided in the Supplementary Materials.

### 2.3 Downstream applications

#### 2.3.1 Drug response prediction

**Sensitive Drug Classification.** A cross-drug dataset containing seven representative cell lines (A375, PC3, MCF7, HT29, HeLa, YAPC, and HA1E) and 335 drugs was constructed for training and evaluation. The trained perturbation models were subsequently applied to predict perturbed expression for an additional 7148 previously unseen drugs (see Table S3).

Drug sensitivity annotations AUC (area under the dose-response curve) values were obtained from the Cancer Therapeutics Response Portal (CTRP), covering four cell lines (A375, PC3, MCF7, and HT29), 267 of the unseen drugs and 803 drug-cell combinations (Table S5). Smaller AUC means greater cell sensitivity (AUC values in CTRP range from 2.6 to 18.6) and we set AUC < 5.5 as sensitivity following TranSiGen (Tong *et al.* 2024).

Each model (TranSiGen, PertDiff-no, PertDiff) generated perturbed gene expression data, from which differentially expressed genes (DEGs) were calculated as the difference between perturbed and control transcriptomes. These DEGs features were then used to train independent random forest (RF) classifiers to predict binary drug sensitivity labels.

Drugs were divided into training (80%) and testing (20%) sets without overlap. All experiments were repeated five times with distinct random seeds. Model performance was evaluated using the area under the receiver operating characteristic (ROC) curve (AUC), which quantifies the ability to discriminate between sensitive and resistant drugs.

**Cellular Drug Response.** To evaluate drug response prediction at the cellular level, perturbation dataset was aligned with the GDSC2 database, yielding 14 overlapping cell lines and 273 drugs. After filtering drugs without valid SMILES representations and resolving duplicated measurements, the final dataset contained 14 cell lines, 114 drugs, and 1483 drug–cell combinations.

Perturbation models were trained using the remaining cell lines without IC_50_ annotations to avoid information leakage (see Table S3). Predicted perturbed expression profiles were then generated for each drug-cell combination in the evaluation set, and differential expression vectors were derived relative to control expression.

These features were used to train Random Forest regressors to predict log-transformed IC_50_ values (LN_IC_50_). Cell lines were split into training (80%) and testing (20%) subsets, and experiments were repeated five times with different random seeds. Model performance was assessed using Pearson correlation coefficients and prediction error distributions. Detailed dataset filtering and model training procedures are described in the Supplementary Materials.

#### 2.3.2 Drug repurposing

We followed the same candidate set and data sources as TranSiGen. In brief, 1625 candidate drugs were considered for pancreatic cancer repurposing; among which 432 drugs had available AUC(area under the dose-response curve) values from the PRISM dataset (Corsello *et al.* 2020).

Using the model trained under the cross-drug scenario, we predicted transcriptional responses of the pancreatic cancer cell line to all 1625 drugs. Predicted expression profiles were expanded from 978 landmark genes to a full set of 10 174 genes by including 9196 best-inferred genes (Subramanian *et al.* 2017).

The disease query signature for the YAPC cell line was derived from the TCGA-PAAD cohort and defined as genes differentially expressed between pancreatic tumor and normal tissues, consisting of 293 up-regulated genes ($S_{up}$) and 168 down-regulated genes ($S_{\mathrm{down}}$). These gene sets (${|S}_{up}|=293$, ${|S}_{\mathrm{down}}|=168$) were used for enrichment and connectivity scoring against each drug’s predicted ranked gene list. For each drug instance, the n=10 174 genes were ranked by predicted DEGs in descending order. Let V(j) be the rank position of the j-th gene in a query set when mapped into the drug’s ranked list. Enrichment scores for $S_{up}$ and $S_{\mathrm{down}}$ using a Kolmogorov–Smirnov statistic. For a query set S (size t):


(9) a=maxj=1 to t[jt-V(j)n]

(10) $$b=\underset{j=1\;\mathrm{to}\;t}{\max}\left[\frac{V(j)}{n}-\frac{j-1}{t}\right]$$

The enrichment score (ES) for S is then:


(11) ESupdown={aupdown(if aupdown>bupdown)-bupdown(if aupdown<bupdown)

Thus ${ES}_{up}$ and ${ES}_{\mathrm{down}}$ measure whether the disease up-genes and down-genes are enriched at the top (positive ES) or bottom (negative ES) of the drug-ranked list. If ${ES}_{up}$ and ${ES}_{\mathrm{down}}$ share the same sign, indicating concordant changes with the disease state, the connectivity score is set to zero. Otherwise, the connectivity score (CS) is defined as:


(12) CS=ESup-ESdown

CS belongs to [−1, 1], 1 indicates maximal concordance (drug enhances the disease signature), while -1 indicates maximal reversal of the disease signature. Each drug was scored in three random seeds, and the mean CS across seeds was used for final ranking; drugs with scores close to -1 were considered high-priority reversal candidates.

For literature validation, PRISM AUC values were used to define a label set of drugs: among the 432 drugs with available PRISM AUCs, drugs in the bottom 4th percentile of AUC (AUC values in PRISM range from 0 to 2; the 4th percentile corresponds to AUC < 0.5, which is the 17th smallest among 432 drugs) were labeled as known sensitive (positive class, label = 1). Manual literature verification was limited to top-ranked candidates appearing before the first labeled positive in the ranked list.

#### 2.3.3 Clinical data validation

**Data Source and Preprocessing.** Three independent GEO cohorts of breast cancer patients treated with paclitaxel were used, GSE25055, GSE32646, and GSE20194. Samples were annotated as pathological complete response (pCR) or residual disease (RD), where pCR indicates strong therapeutic response while RD represents incomplete remission (Jia *et al.* 2021). Control gene expression profiles were aligned to the 978 landmark genes used as input to the PertDiff model.

**Perturbation Inference.** Using the model trained under the cross-cell line scenario, we predicted perturbed expression for each patient from their control expression and the molecular feature of paclitaxel. Paclitaxel’s SMILES string was retrieved from DrugBank and cell line description was derived from GEO.

**Quantifying Perturbation Magnitude.** To quantify predicted transcriptional responses, we computed energy distances (E-distance) (Peidli *et al.* 2024) between control and predicted perturbed expression distributions. After resampling RD and pCR cohorts to equal sizes, expression profiles (both control and predicted) were projected onto the top principal components derived from the combined dataset. The number of principal components retained was adjusted per cohort based on the total number of samples (Table S8). E-distance values were then calculated between control and perturbed samples within each label (pCR or RD). E‑distance measures the statistical distance between two distributions. Larger E-distance values indicate greater transcriptional divergence and stronger predicted drug responses. Mathematical definitions and implementation details are provided in the Supplementary Materials. All reported E‑distances are averaged across five independent runs with different random seeds, and statistical significance between pCR and RD groups was assessed using two‑tailed paired *t*‑tests.

## 3 Results

### 3.1 Overview

PertDiff is a multimodal conditional diffusion framework designed to predict drug-induced transcriptional perturbations by integrating heterogeneous biological priors. Built upon a DDPM backbone (Fig. 1A), PertDiff employs a sophisticated conditioning mechanism to integrate control gene expression with chemical and biological contexts. Specifically, drug molecular structures are encoded via a pretrained graph neural network (MolGNet), while cell line characteristics are captured through semantic embeddings of LLM-generated descriptions. These modality-specific features are fused within a conditional network, where cross-attention mechanisms enable cell-context-aware modulation of the generative process. Through this architecture, PertDiff progressively denoises latent variables to construct biologically coherent perturbed gene expression profiles.

To rigorously assess the model’s capabilities, we benchmarked PertDiff against state-of-the-art methods under challenging cross-drug and cross-cell settings, evaluating both prediction accuracy and generalization. We further conducted ablation studies to quantify the contribution of integrating text-based cell descriptions. Finally, to demonstrate that the generated perturbations preserve authentic pharmacological signals, we validated the model across diverse downstream applications, including drug response prediction, drug repurposing, and clinical validation (Fig. 1B).

### 3.2 PertDiff outperforms existing models across drugs and cell lines

**Figure 2:**
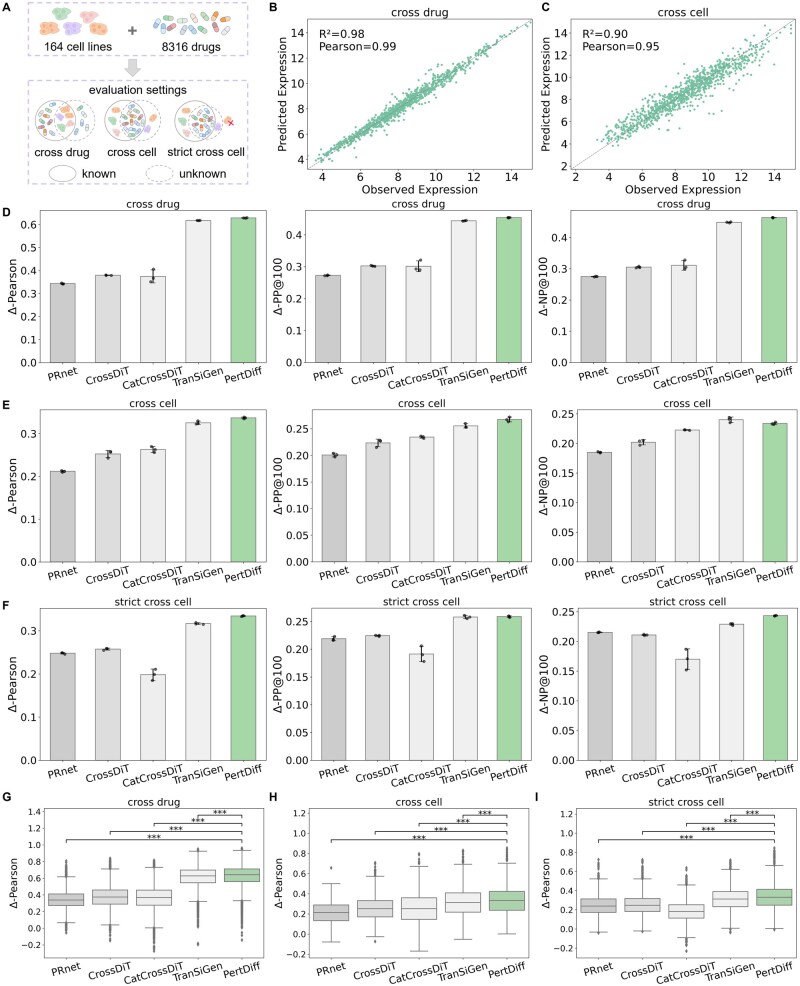
Evaluation settings and model performance comparison. (A) Schematic illustration of the three evaluation scenarios applied to 164 cell lines and 8316 drugs. Different colors represent distinct cell lines and drugs. In the cross drug scenario, training and test sets share the same cell lines but involve different drugs. In the cross cell scenario, the same drugs are used, but training and test sets consist of different cell lines. The strict cross cell scenario further increases the dissimilarity between training and test cell lines. Specifically, the training set includes PC3, HT29, A375, HEPG2, HCC515, YAPC, HS578T, BT20, HA1E, and JURKAT; the validation set includes SKBR3 and MCF7; and the test set includes A549, HeLa, and MDAMB231. ‘Known’ refers to drug-cell combinations observed during training, while ‘unknown’ denotes previously unseen combinations. (B) Scatter plot comparing predicted versus observed gene expression under cross drug. (C) Scatter plot comparing predicted versus observed gene expression under cross cell. Each plot displays a randomly selected drug-cell pair from the respective test set, annotated with its R^2^ and Pearson correlation coefficient. (D) Performance comparisons across models in cross drug scenario. (E) Performance comparisons across models in cross cell scenario. (F) Performance comparisons across models in strict cross cell scenario. Bars represent mean values of three metrics (Δ-Pearson, Δ-PP@100, and Δ-NP@100) across three independent runs with different random seeds. Error bars indicate standard deviation. Individual run results are shown as black dots. (G) Boxplots showing the distribution of model performance for cross drug. (H) Boxplots showing the distribution of model performance for cross cell. (I) Boxplots showing the distribution of model performance for strict cross cell. Each boxplot shows the distribution of model predictions from one run (the same random seed was used across models for this comparison plot), illustrating interquartile range, median, and outliers. Statistical significance was assessed using two-tailed paired t-tests. Significance levels: *P < 0.05, **P < 0.01, ***P < 0.001, ns = not significant.

To systematically evaluate PertDiff’s predictive performance, we benchmarked it against four state-of-the-art methods, Prnet (Qi *et al.* 2024), CrossDiT, CatCrossDiT [both from Hu *et al.* (2026)], and TranSiGen (Tong *et al.* 2024), using a dataset of 164 cell lines and 8316 drugs. As shown in Fig. 2A, three hierarchical generalization settings were designed: cross drug (unseen drugs with fixed cell lines), cross cell (unseen cell lines with fixed drugs), and strict cross cell [distinct cell line clusters with minimal contextual overlap (Wu *et al.* 2022)]. All methods were evaluated on the same splits per scenario (see Table S3).

**Cross Drug Scenario.** In the cross drug setting, PertDiff accurately captured transcriptional structures. The scatter plot of predicted versus observed gene expression (Fig. 2B) shows a strong correlation (R^2^ = 0.98, Pearson = 0.99) for a drug-cell pair. Performance on DEGs (Δ = perturbed—control gene expression) was evaluated using three distinct metrics (Fig. 2D): Δ-Pearson to measure overall correlation, and Δ-PP@100 and Δ-NP@100 to evaluate the accuracy in retrieving the top 100 upregulated and downregulated genes, respectively. PertDiff achieved the highest scores across all methods, with a Δ-Pearson of 0.629, a Δ-PP@100 of 0.454, and a Δ-NP@100 of 0.464 (Table S1). Notably, PertDiff surpassed the second-best model, TranSiGen, by 10.2% in R^2^. The distribution of performance across samples (Fig. 2G) confirms this superiority; PertDiff exhibits a higher median Δ-Pearson and statistically improvement over all baseline models, highlighting its robustness in predicting responses to novel drugs.

**Cross Cell Scenario.** Predicting responses in unseen cellular contexts is more challenging due to biological heterogeneity, yet PertDiff maintained strong performance. The scatter plot (Fig. 2C) shows a high correlation (R^2^=0.90). As shown in Fig. 2E, PertDiff remained the top-performing method across metrics. It achieved a Δ-Pearson of 0.337, outperforming TranSiGen (0.326). Crucially, PertDiff demonstrated superior capability in identifying significant gene expression changes, achieving the highest scores in Δ-PP@100 (0.267). This indicates that even when exact numerical prediction becomes difficult in new cell lines, PertDiff remains superior at correctly prioritizing the most significant regulatory changes in upregulated genes. The boxplot analysis (Fig. 2H) further validates these results, showing that PertDiff achieves a higher distribution of correlation scores compared to competing models.

**Strict Cross Cell Scenario.** To rigorously probe model generalization, we employed the strict cross-cell split using fold 2 of the gene-expression-based five-fold cross-validation from Wu *et al.* (2022) (cell line assignment in Fig. 2 caption). As expected, this rigorous setting caused a general performance drop across all models (Table S1). However, PertDiff exhibited the highest resilience (Fig. 2F), achieving the best results in global correlation (Δ-Pearson: 0.334) as well as gene set retrieval (Δ-PP@100 = 0.259 and Δ-NP@100 = 0.243). The boxplots in Fig. 2I demonstrate that even under these stringent conditions, PertDiff consistently outperforms baselines, confirming its ability to learn transferable biological principles rather than merely memorizing cell-specific patterns.

Overall, PertDiff consistently outperforms state-of-the-art methods across increasingly challenging scenarios. Its superior performance in both global correlation (Δ-Pearson) and key gene retrieval (Δ-PP@100, Δ-NP@100), validated by statistical significance across all settings (Fig. 2G–I), demonstrates strong generalization and suitability for robust drug–cell perturbation prediction.

### 3.3 Incorporating cell description enhances prediction performance and biological relevance

**Figure 3:**
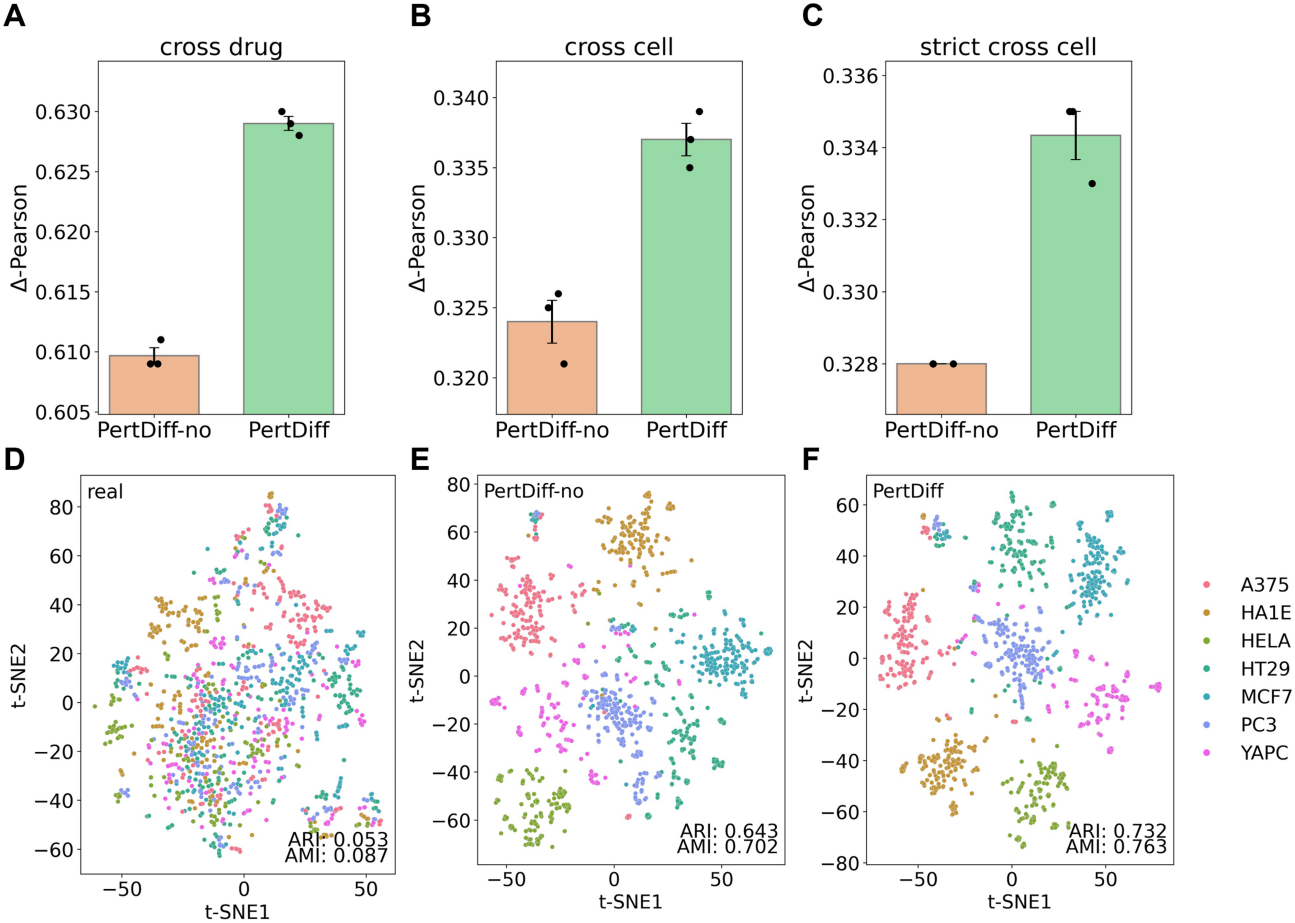
Comparative analysis of PertDiff-no and PertDiff models in gene expression prediction. (A) Cross-drug scenario: Bar plot comparing the mean Pearson correlation between predicted and observed Δ-expression for PertDiff-no and PertDiff. Error bars denote standard deviation across three random seeds. Individual run results are marked as black dots. (B) Cross-cell scenario: Same as (A), showing performance in the cross-cell evaluation. (C) Strict cross-cell scenario: Same as (A), showing performance in the strict cross-cell evaluation. (D) t-SNE embeddings of experimental Δ-expression profiles: Each point represents a drug–cell combination, colored by true cell line identity (A375, HA1E, HeLa, HT29, MCF7, PC3, and YAPC). Adjusted Rand Index and Adjusted Mutual Information values in the bottom-right corner quantify clustering alignment with observed cell line labels. (E) t-SNE embeddings of PertDiff-no predictions: Same as (D), but for Δ-expression profiles predicted by PertDiff-no. (F) t-SNE embeddings of PertDiff predictions: Same as (D), but for Δ-expression profiles predicted by PertDiff. t-SNE-reduced Δ-expression profiles were clustered with K-means (K = 7), and ARI/AMI scores were calculated to evaluate cluster alignment with true cell line identities.

To quantify the contribution of cell line descriptions to the model’s predictive capability, we conducted an ablation study comparing the full PertDiff model against a variant excluding cell descriptions (PertDiff-no), while keeping all other architectures identical. As presented in Table S2 and Fig. 3A–C, the incorporation of cell descriptions consistently improves prediction accuracy across all three evaluation scenarios. In the cross-drug setting, PertDiff demonstrated gains, with the R^2^ increasing by 10.57%, the RMSE decreasing by 2.46%, and the Pearson correlation of Δ increasing by 3.11% (Fig. 3A). Similarly, in the cross-cell line setting, the model achieved a 3.89% improvement in PP@100 and a 4.01% increase in Pearson correlation (Fig. 3B). Even in the most challenging strict cross-cell line setting, where the model encounters entirely unseen cell lines, the inclusion of textual descriptions yielded a 1.83% increase in Pearson correlation (Fig. 3C). These quantitative metrics validate that semantic descriptions serve as a crucial prior, guiding the model to generalize better across different biological contexts.

To evaluate biological heterogeneity, we visualized DEGs using t-distributed stochastic neighbor embedding (t-SNE) and quantified the alignment of the resulting clusters with the observed cell-line identities using Adjusted Rand Index (ARI) and Adjusted Mutual Information (AMI) metrics (Maaten and Hinton 2008, Romano *et al.* 2016). We observed that the experimental DEGs exhibit a highly entangled distribution with negligible clustering by cell line (Fig. 3D; ARI: 0.053). In contrast, the predicted DEGs reveal distinct cell-specific structures. While PertDiff-no forms observable clusters (Fig. 3E; ARI: 0.643), the PertDiff model achieves better separation and compactness (Fig. 3F; ARI: 0.732). This demonstrates that incorporating cell descriptions enables the model to effectively recover and preserve cell-specific characteristics.

### 3.4 PertDiff enables accurate sensitive drugs classification and response prediction

**Figure 4:**
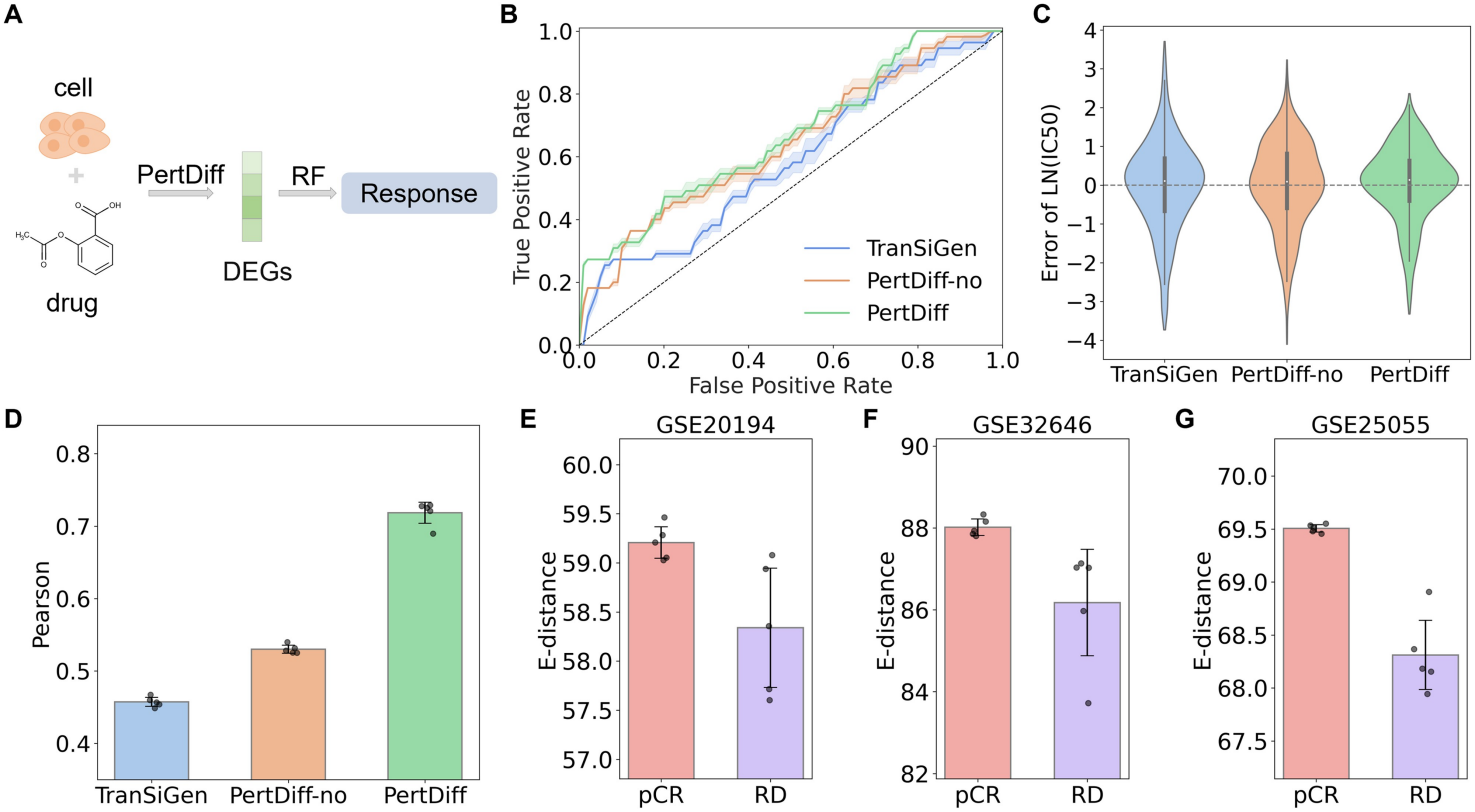
Drug response prediction under cross drug and cross cell settings and clinical validation on three GEO datasets. (A) Workflow overview of the drug response prediction pipeline. The perturbation model generates predicted perturbed gene expression profiles, from which DEG signatures are extracted and used to train a RF for predicting drug sensitivity response. Performance is evaluated on an unseen test set. (B) ROC curves showing the predictive performance of the RF-classifier model under the cross drug setting. (C) Violin plots depicting the distribution of errors between observed and predicted ln(IC_50_) values under the cross cell setting. Smaller absolute error indicates more accurate ln(IC_50_) prediction. (D) Bar plots showing the Pearson correlation coefficient between predicted and observed ln(IC_50_) values under the cross cell setting. (E–G) Clinical validation on three independent breast cancer datasets from GEO. Bar plots compare the mean E-distance between pCR and RD samples. Individual run results are marked as black dots. Error bars denote standard deviation across five random seeds.

PertDiff is designed to predict drug-induced transcriptional perturbations in cells, capturing the intricate effects of drugs on cellular states. Beyond this primary task, its predictions can be leveraged to classify drug sensitivity and to estimate cellular responses such as IC_50_. This downstream application builds on the biological principle that DEGs encode drug sensitivity: DEGs associated with sensitive drugs tend to be more similar to one another than to those associated with resistant drugs (Tong *et al.* 2024). Guided by this principle, we constructed an application framework in which PertDiff-predicted DEGs are used as high-dimensional features for Random Forest models to predict phenotypic outcomes (Fig. 4A).

**Sensitive Drugs Classification.** We first evaluated the utility of PertDiff for drug prioritization by determining whether a cell line is sensitive or resistant to unseen drugs. Using the CTRP dataset (Table S5), which includes 803 cell line-drug pairs, we defined sensitivity based on dose-response AUC values. In this cross-drug setting, the RF-classifier trained on PertDiff-predicted features demonstrated superior predictive power compared to baselines. The ROC curves (Fig. 4B) indicate that the transcriptional perturbations predicted by PertDiff robustly encode drug-sensitivity signals, enabling accurate classification even for chemically diverse agents not encountered during training.

**Cellular Response IC_50_ regression.** Moving beyond binary classification, we assessed whether the model could capture quantitative variations in drug efficacy across genetically distinct tumor cell lines. Focusing on the cross-cell setting, we utilized the GDSC2 dataset (Table S7) to train a RF-regressor to map predicted DEGs to log-transformed IC_50_ values for 1483 drug-cell combinations. The results confirm that PertDiff preserves subtle, context-dependent biological signals. As shown in Fig. 4C, the distribution of prediction errors for PertDiff is more centered around zero compared to other methods, reflecting higher precision. Furthermore, the model achieved a Pearson correlation coefficient of 0.718 between predicted and observed log-transformed IC_50_ values (Fig. 4D). These metrics demonstrate that PertDiff successfully disentangles how cellular heterogeneity modulates treatment outcomes, providing a reliable surrogate for experimental screening.

### 3.5 Identifying therapeutic candidates for pancreatic cancer via drug repurposing

Drug repurposing offers an efficient strategy to identify new therapeutic opportunities from existing drugs, particularly for malignancies with limited treatment options. Pancreatic cancer remains one of the most lethal solid tumors, characterized by strong molecular heterogeneity and resistance to chemotherapy (Neoptolemos *et al.* 2018). Motivated by the role of transcriptional dysregulation in tumor progression and drug response (Bailey *et al.* 2016), we aimed to identify drugs capable of reversing the pancreatic cancer transcriptional signature.

To systematically screen for such candidates, we applied PertDiff within the Connectivity Map framework (Lamb *et al.* 2006), which prioritizes drugs that induce transcriptional changes opposite to disease signatures. A disease query signature was constructed from genes differentially expressed between pancreatic tumors (TCGA-PAAD) and normal tissue, and matched against PertDiff-predicted perturbation profiles of 1625 PRISM drugs (Corsello *et al.* 2020). Connectivity scores quantified the extent to which drugs reverse the disease signature. As summarized in Table 1, this screening showed high predictive validity: 10 of the top 17 ranked drugs have literature-supported anti-pancreatic cancer activity.

**Table 1:** Summary of literature-based validation for prioritized drug candidates.

Rank	Drug name	Ref.	Mechanism of action
1	Flurothyl	None	None
2	SB-939	(Lomberk *et al.* 2016)	HDAC inhibitor
3	Trichostatin-a	(Lomberk *et al.* 2016)	HDAC inhibitor
4	Rolziracetam	None	None
5	Panobinostat	(Lomberk *et al.* 2016)	HDAC inhibitor
6	Gambogic-acid	(Youns *et al.* 2018, Wang *et al.* 2019)	Caspase activator
7	Dacinostat	(Lomberk *et al.* 2016)	HDAC inhibitor
8	Belinostat	(Lomberk *et al.* 2016)	HDAC inhibitor
9	Thiostrepton	(Zhang *et al.* 2022)	FOXM1 inhibitor, protein synthesis inhibitor
10	Scopine	None	None
11	Etebenecid	None	Uricosuric blocker
12	3,3'-dichlorobenzaldazine	None	Glutamate receptor allosteric ligand
13	CPCCOEt	None	Glutamate receptor antagonist
14	BNTX	(Kim *et al.* 2017)	Opioid receptor antagonist
15	Resminostat	(Lomberk *et al.* 2016)	HDAC inhibitor
16	Meparfylon	None	None
17	Peruvoside	(Feng *et al.* 2016)	Cardiac glycoside

Specifically, gambogic acid, traditionally used as an anti-inflammatory and hemostatic agent, has been reported to inhibit pancreatic cancer cell growth. Its mechanism involves up-regulating stress and apoptosis markers (e.g. DDIT3, DUSP1) and promoting ROS accumulation and autophagy (Youns *et al.* 2018, Wang *et al.* 2019). Thiostrepton, originally approved as a veterinary antibiotic, has been reported to inhibit pancreatic cancer by blocking STAT3/GPX4 signaling and inducing ferroptosis (Zhang *et al.* 2022). BNTX, a δ-opioid receptor antagonist used for pain modulation, has been shown to suppress pancreatic cancer by enhancing TRAIL-induced apoptosis through PKCα/AKT inhibition and XIAP degradation (Kim *et al.* 2017, Wang *et al.* 2024). Peruvoside, a cardiac glycoside for heart failure, has been reported to reduce the survival, proliferation, and migration of pancreatic cancer cells (Feng *et al.* 2016). Notably, histone deacetylase (HDAC) inhibitors were frequently top-ranked, suggesting HDAC inhibition as a convergent therapeutic mechanism in pancreatic cancer (Lomberk *et al.* 2016). Top-ranked drugs without literature support (e.g. Flurothyl, Rolziracetam) represent candidate that await experimental validation.

Together, these results show that PertDiff can effectively prioritize therapeutically relevant candidates by identifying drugs that reverse disease transcriptional programs, providing a scalable approach for repurposing existing drugs in precision oncology.

### 3.6 Predicted perturbations reflect real-world drug responses in clinical data

To assess whether PertDiff-predicted perturbations align with clinical drug responses, we validated PertDiff on three independent GEO cohorts of paclitaxel-treated breast cancer patients [GSE20194 (pCR, n = 56; RD, n = 222), GSE32646 (pCR, n = 27; RD, n = 88), and GSE25055 (pCR, n = 122; RD, n = 188)]. Here, pCR denotes pathological complete response, while RD represents residual disease; pCR samples typically exhibit stronger drug-induced perturbation effects. Across all cohorts, pCR samples consistently showed larger predicted E-distances than RD samples (Fig. 4E–G), indicating stronger inferred perturbations in clinically responsive patients. For GSE20194, the mean E‑distance was 59.21 for pCR and 58.34 for RD (*P* = 0.029, two‑tailed *t*‑test); for GSE25055, 69.51 and 68.31 (*P* = 0.0017); and for GSE32646, 88.02 and 86.18 (*P* = 0.041). These results indicate that PertDiff captures clinically relevant drug-response signals, predicting stronger transcriptional perturbations in patients who respond well to paclitaxel, and suggesting potential utility for patient-level drug sensitivity prediction from baseline gene expression.

## 4 Discussion

We introduced PertDiff, a conditional diffusion framework that integrates control gene expression, molecular structures, and semantic cell descriptions to model cellular responses. PertDiff generates biologically coherent perturbations and demonstrates robust generalization to unseen drugs and cell lines, providing a solid foundation for downstream tasks such as drug response prediction, drug repurposing, and clinical validation.

The repurposing workflow established here offers a scalable strategy applicable to diverse disease contexts. For diseases characterized by heterogeneity, results from multiple corresponding cell lines can be integrated to identify consensus therapeutic candidates with higher confidence. Furthermore, while current screening strategies primarily focus on reversing transcriptional signatures, our results indicate a meaningful correlation between DEGs and cellular phenotypes, such as IC_50_ and AUC. Consequently, a holistic screening approach that simultaneously incorporates gene-level reversal signals and phenotype-level predictive outcomes holds promise for more precisely prioritizing effective drugs.

While PertDiff achieves state-of-the-art performance, accurately modeling perturbations across cell lines remains a challenge in the field, largely due to the intrinsic properties of transcriptomic data. The high dimensionality of the transcriptome (978 genes), contrasted with the finite number of available cell lines (164 cell lines), creates a sparse data landscape that challenges the extraction of universal patterns. Moreover, unlike static chemical structures, cellular systems are inherently dynamic; variations arising from cell-cycle fluctuations and experimental batch effects introduce intrinsic stochasticity. These factors underscore the complexity of the biological ground truth, suggesting that future improvements will scale with the use of more comprehensive datasets. Additionally, while LLM-derived cell descriptions improve generalization, they may inherit biases from their training corpora; future work could explore learning cell representations directly from multi-omic data.

Looking forward, PertDiff serves as an extensible framework to address these complexities. Future iterations will aim to adapt the model to single-cell data, enabling higher-resolution capture of cellular heterogeneity. Additionally, while the current study focuses on single drugs, clinical oncology practice often relies on combination therapies to overcome resistance. We envision extending PertDiff with modules designed to learn non-linear drug interactions, thereby facilitating the prediction of synergistic effects and broadening the scope of in silico therapeutic discovery.

## Supplementary Material

btag482_Supplementary_Data
