# Supplementary material for: Enhancing cross-context generalization in drug perturbation prediction with a multimodal conditional diffusion framework
Source: Bioinformatics. 2026 Jun 30;42(7):btag482. doi: 10.1093/bioinformatics/btag482 (PMC13364675; doi:10.1093/bioinformatics/btag482)
Supplement: btag482_Supplementary_Data [file btag482_supplementary_data.zip › supplementary_materials.docx]

**Supplementary Materials for Enhancing Cross-Context Generalization in Drug Perturbation Prediction with a Multimodal Conditional Diffusion Framework**

Yanjie Ma^1^, Kang Du^1^, Yan Li^1, 2, *^, Pengyong Li^1, *^, and Liang Yu^1, *^

^1^School of Computer Science and Technology, Xidian University, Xi’an 710126, Shaanxi, China.

^2^School of management, Xi'an Polytechnic University, Xi’an 710000, Shaanxi, China.

^*^Corresponding author. Email: [lyu@xidian.edu.cn](mailto:lyu@xidian.edu.cn), [lipengyong@xidian.edu.cn](mailto:lipengyong@xidian.edu.cn), [20170914@xpu.edu.cn](mailto:20170914@xpu.edu.cn).

**Supplementary Materials for Data Source and Feature Construction**

**Gene Expression Profiles.** The gene expression data were derived from the CMAP LINCS 2020 dataset released by the Broad Institute, one of the largest publicly available datasets of perturbational transcriptomic profiles(Institute, 2020; Lamb, et al., 2006; Subramanian, et al., 2017). The dataset, generated using the L1000 platform, measures 978 landmark genes and infers 9,196 additional genes, resulting in over one million perturbational profiles across diverse drugs, genetic interventions, concentrations, time points, and cell lines(Subramanian, et al., 2017). We adopted the processed level 3 data provided by the TranSiGen study, selecting samples with 10 μM drug concentration and 24 h treatment(Tong, et al., 2024). To reduce batch effects, perturbed samples were paired with controls within the same plate, and replicates were aggregated using the MODZ algorithm. After preprocessing, 78,569 drug-cell combinations covering 164 cell lines and 8,316 drugs were obtained, each containing control and perturbed expression of 978 genes. For computational efficiency, gene expression values were linearly transformed so that the majority of data fall within the range [-1, 1].

**Drug Representations.** Drug molecular features were extracted using the pre-trained MolGNet model(Li, et al., 2021), which was originally trained under the MPG (Masked Property Graph) pre-training framework on millions of unlabeled molecules. MolGNet encodes molecular graphs derived from simplified molecular input line entry system (SMILES) strings and produces graph-level embeddings that capture structural, electronic, and higher-order semantic information. In this study, we directly employed the publicly released MolGNet checkpoint to obtain the graph-level embedding as the molecular representation, without additional fine-tuning.

**Cell Description Feature.** To enrich cell line representations, we incorporated semantic textual features derived from curated cell description. Specifically, the DeepSeek model was first employed to generate descriptive summaries for each cell line, including its tissue origin, related diseases, highly expressed genes, and known therapeutics(DeepSeek, 2025). The extracted description was then refined using the ChatGPT model to enhance clarity, consistency, and biological relevance, followed by manual verification to ensure factual accuracy(OpenAI, 2025). Finally, each curated description was encoded into a 384-dimensional semantic feature using a MiniLM-based Sentence-Transformers model, with mean pooling applied to the last hidden layer to handle variable description lengths(Reimers and Gurevych, 2019). The resulting feature was integrated with gene expression in predictive model to provide biologically meaningful and semantically consistent representations.

The exact prompt templates used to generate the cell line descriptions in Supplementary Note 1. For each cell line, we provided the LLM with the following structured prompts:

DeepSeek (Prompt 1): "Please provide background information about the {} cell line, including which genes are highly expressed, what diseases this cell line corresponds to, what optional drugs are available, and any other information you think is important. Please generate a descriptive text and not segmented (<150 words). Please answer in English."

ChatGPT (Prompt 2): "Whether the above description is correct or not, please correct it if it is wrong. Finally, the output is condensed in English and contains key information."

The generated descriptions were then manually verified for factual accuracy. This information is now included in the Supplementary Materials.

**Supplementary Materials for Implementation Details**

**Pretraining Strategy.** We first pretrain the encoder and decoder modules only for 10,000 epochs, during which the conditional branches are omitted (the right-side conditional network and the left-side cross-attention blocks). To increase the effective pretraining data, noisy inputs $x_{t}$ are formed from both control and perturbed expression and fed through the model’s MLP; no attention or external conditioning is used. The network is trained to predict the clean expression ${\hat{x}_{\theta}(x}_{t},t)$ and optimized with the mean-squared error (MSE) loss:

$$\begin{aligned} L_{MSE}=\frac{1}{D}\sum_{i=1}^{D} \left( {\hat{x}_{\theta,i}(x}_{t},t)-x_{0,i} \right)^{2}\#\left( 1 \right) \end{aligned}$$

where $D=978$ is the number of genes, and ${\hat{x}_{\theta,i}(x}_{t},t)$​ and $x_{0,i}$​ denote the predicted and observed gene values for gene $i$. Parameters are updated by standard backpropagation.

**Fine-tuning and Optimization.** In the fine-tuning stage the conditional network and cross-attention modules are added so that the model receives control expression, drug and cell description features as conditioning signals.

A smaller learning rate, combined with a two-phase decay schedule, is used during fine-tuning to improve stability: for the first 90% of fine-tuning iterations the learning rate is decreased linearly, and during the final 10% a cosine-annealing schedule is applied. During fine-tuning the model is trained with inputs consisting of the noisy gene expression plus the conditioned signals fused via the conditional network and cross-attention blocks to guide the denoising trajectory toward biologically consistent perturbation predictions(Glorot and Bengio, 2010).

**Regularization Strategies.** To mitigate overfitting, we applied weight decay and dropout throughout training. During pretraining, weight decay was 1e−5 and dropout rate 0.1. During fine‑tuning, weight decay was reduced to 1e−6, and dropout rates were set to 0.1 for cross‑drug and cross‑cell settings, 0.5 for the strict cross‑cell setting, 0.3 for sensitive drug classification (cross‑drug), and 0.13 for IC_50_ regression (cross‑cell).

**Parameter Initialization.** Positional encodings are initialized from $N(0,0.02)$; learnable zero-convolution layers are initialized to all zeros; weights of the encoder/decoder that participated in pretraining are initialized from the pretrained checkpoint and frozen; the copied conditional encoder is initialized from the corresponding pretrained parameters but remains learnable; all remaining parameters are initialized with Xavier uniform initialization (Glorot and Bengio, 2010).

**Supplementary Materials for Cellular Drug Response.** We aligned our perturbation dataset with the Genomics of Drug Sensitivity in Cancer (GDSC2) database, identifying 14 overlapping cell lines(5637, A204, A375, AGS, BEN, J82, MCF7, MKN45, OVTOKO, RCC10RGB, YAPC, 22RV1, A549, T47D) and 273 drugs(Yang, et al., 2012). Only 134 drugs with valid SMILES representations obtained from DrugBank were retained.

To reduce batch effects, analyses were restricted to the COMPANY_1046 subset, which contained the largest number of drug–cell combinations with available IC_50_ measurements. For duplicated entries, the lowest IC_50_ value was retained to represent maximal drug sensitivity. Two invalid SMILES that failed RDKit parsing were excluded, resulting in a final dataset of 14 cell lines, 114 drugs, and 1,483 valid drug-cell combinations.

Perturbation models (TranSiGen, PertDiff-no, PertDiff) were trained on the remaining 150 cell lines (without IC_50_ annotations) using a cell-level split strategy to ensure that no cell line appeared in both training and testing sets. For each of the 14 held-out cell lines, perturbed expression profiles were inferred for all 1,483 drug–cell combinations, and DEGs vectors were derived by subtracting control expression from the predicted perturbed expression.

These DEGs features were then used to train RF regressors to predict log-transformed IC_50_ (LN_IC_50_) values. Cell lines were split into training (80%) and testing (20%) subsets, and experiments were repeated five times using different random seeds. Model evaluation included two complementary analyses: violin plots illustrating the prediction errors, and bar plots showing Pearson correlation coefficients between predicted and observed LN_IC_50_ values, reflecting both accuracy and consistency.

**Supplementary Materials for Clinical Data Validation**

**Quantifying Perturbation Magnitude.** To quantify perturbation magnitude, we calculated energy distances (E-distance) between control and perturbed expression profiles. Following resampling of both RD and pCR groups to the same sample size, all samples were projected onto the top 50 principal components computed from the combined dataset. Separate E-distance measurements were then derived for RD and pCR cohorts by comparing their respective control and perturbed groups within this principal component space. The E-distance defined as(Peidli, et al., 2024):

$$\begin{aligned} E\left( X,Y \right):=2\delta_{XY}-\sigma_{X}-\sigma_{Y}\#\left( 2 \right) \end{aligned}$$

where

$$\begin{aligned} \sigma_{X}=\frac{1}{N^{2}}\sum_{i=1}^{N} \sum_{j=1}^{N} \left\| x_{i}-x_{j} \right\|\#\left( 3 \right) \end{aligned}$$

$$\begin{aligned} \sigma_{Y}=\frac{1}{M^{2}}\sum_{i=1}^{M} \sum_{j=1}^{M} \left\| y_{i}-y_{j} \right\|\#\left( 4 \right) \end{aligned}$$

$$\begin{aligned} \delta_{XY}=\frac{1}{NM}\sum_{i=1}^{N} \sum_{j=1}^{M} \left\| x_{i}-y_{j} \right\|\#\left( 5 \right) \end{aligned}$$

Here, $X=\left\{ x_{i} \right\}_{i=1}^{N}$ and $Y=\left\{ y_{i} \right\}_{i=1}^{M}$ represent the sets of control and predicted perturbed expression, respectively. $\sigma_{X}$ and $\sigma_{Y}$ measure the within-group compactness of control and perturbed samples, while $\delta_{XY}$ measures the cross-group distance between control and perturbed expression. The E-distance $E\left( X,Y \right)$ therefore captures how distinct the two groups are: larger values indicate a greater transcriptional divergence induced by drug perturbation, which in this study corresponds to a stronger predicted drug response.

**Supplementary Materials for Evaluation Metrics:**

Model performance was assessed using four categories of metrics: Pearson correlation, Precision@100, R², and RMSE, computed across all cell-drug combinations in the test set.

**Pearson correlation** quantified the agreement between predicted and observed expression profiles. For each drug-cell combination, 978-dimensional vectors of predicted ($x'$) and observed ($x$) perturbation expression were compared:

$$\begin{aligned} Pearson\left( x\sim x^{'} \right)=\frac{\sum_{i=1}^{978} \left( x_{i}-\bar{x} \right)\left( {x^{'}}_{i}-\bar{x^{'}} \right)}{\sqrt{\sum_{i=1}^{978} \left( x_{i}-\bar{x} \right)^{2}\sum_{i=1}^{978} \left( {x^{'}}_{i}-\bar{x^{'}} \right)^{2}}}\#(6) \end{aligned}$$

Similarly, Pearson(Δ~Δ′) was computed for differential expression vectors($\Delta=x_{perturbed}-x_{control}$). The final Pearson score was averaged over all combinations.

**Precision@100** measured the accuracy of predicting the top 100 most perturbed genes. PP@100 and NP@100 were calculated as the proportion of overlap between the predicted and observed top 100 upregulated or downregulated genes, respectively. The final score was averaged over all combinations.

**Coefficient of determination (R²) and RMSE** were computed per combination using standard formulas for differential expression ($\Delta\sim\Delta'$) and then averaged across the test set:

$$\begin{aligned} R^{2}=1-\frac{\sum_{i=1}^{978} \left( \Delta_{i}-{\Delta^{'}}_{i} \right)^{2}}{\sum_{i=1}^{978} \left( \Delta_{i}-\bar{\Delta} \right)^{2}}\#(7) \end{aligned}$$

$$\begin{aligned} RMSE=\sqrt{\frac{1}{978}\sum_{i=1}^{978} \left( \Delta_{i}-{\Delta^{'}}_{i} \right)^{2}}\#\left( 8 \right) \end{aligned}$$

Higher Pearson and R² values, along with lower RMSE, indicate better predictive performance, while Precision@100 evaluates the model’s ability to capture the most strongly perturbed genes.

**Supplementary Note 1: Prompt templates for LLM-based cell line description generation**

To ensure reproducibility of the cell line description generation process described in Section 2.1, we provide the exact prompt templates used for two large language models (DeepSeek and ChatGPT).

Prompt 1 (DeepSeek): "Please provide background information about the {} cell line, including which genes are highly expressed, what diseases this cell line corresponds to, what optional drugs are available, and any other information you think is important. Please generate a descriptive text and not segmented (<150 words). Please answer in English."

Prompt 2 (ChatGPT): "Whether the above description is correct or not, please correct it if it is wrong. Finally, the output is condensed in English and contains key information."

The placeholder {} was replaced with the name of each cell line (e.g., A375, PC3, MCF7). The output from Prompt 1 was fed as input to Prompt 2, and the final descriptions were manually verified against public resources to confirm factual accuracy.

**Supplementary Table 1.** Performance comparison of different models on the benchmark dataset.

|  |  | PRnet | CrossDiT | CatCrossDiT | TranSiGen | PertDiff |
| --- | --- | --- | --- | --- | --- | --- |
| Cross-drug | Pearson(x~x’)↑ | 0.913±0.001 | 0.946±0.001 | 0.945±0.005 | 0.968±0.000 | **0.969±0.000** |
|  | Pearson(Δ~Δ‘)↑ | 0.343±0.002 | 0.38±0.001 | 0.375±0.028 | 0.618±0.001 | **0.629±0.001** |
|  | PP@100(Δ~Δ‘)↑ | 0.273±0.001 | 0.302±0.002 | 0.301±0.017 | 0.444±0.001 | **0.454±0.001** |
|  | NP@100(Δ~Δ‘)↑ | 0.275±0.001 | 0.305±0.003 | 0.311±0.015 | 0.449±0.002 | **0.464±0.001** |
|  | R^2^(Δ~Δ‘)↑ | -1.192±0.028 | -0.195±0.009 | -0.361±0.255 | 0.332±0.004 | **0.366±0.001** |
|  | RMSE(Δ~Δ‘)↓ | 0.894±0.005 | 0.697±0.002 | 0.731±0.055 | 0.525±0.001 | **0.516±0.000** |
| Cross-cell line | Pearson(x~x’)↑ | 0.790±0.006 | 0.895±0.005 | **0.936±0.002** | 0.902±0.002 | 0.917±0.001 |
|  | Pearson(Δ~Δ‘)↑ | 0.212±0.002 | 0.253±0.008 | 0.263±0.007 | 0.326±0.004 | **0.337±0.002** |
|  | PP@100(Δ~Δ‘)↑ | 0.201±0.004 | 0.223±0.007 | 0.234±0.002 | 0.255±0.004 | **0.267±0.005** |
|  | NP@100(Δ~Δ‘)↑ | 0.185±0.001 | 0.202±0.004 | 0.223±0.001 | **0.240±0.005** | 0.234±0.002 |
|  | R^2^(Δ~Δ‘)↑ | -5.79±0.255 | -1.93±0.164 | **-0.62±0.160** | -1.819±0.045 | -1.323±0.047 |
|  | RMSE(Δ~Δ‘)↓ | 1.445±0.029 | 0.997±0.024 | **0.791±0.032** | 0.951±0.005 | 0.881±0.006 |
| Strict cross-cell line | Pearson(x~x’)↑ | 0.842±0.001 | 0.852±0.002 | 0.902±0.01 | 0.891±0.002 | **0.907±0.001** |
|  | Pearson(Δ~Δ‘)↑ | 0.248±0.002 | 0.258±0.002 | 0.198±0.013 | 0.317±0.002 | **0.334±0.001** |
|  | PP@100(Δ~Δ‘)↑ | 0.219±0.004 | 0.224±0.001 | 0.191±0.014 | 0.258±0.003 | **0.259±0.002** |
|  | NP@100(Δ~Δ‘)↑ | 0.215±0.001 | 0.211±0.001 | 0.17±0.018 | 0.229±0.002 | **0.243±0.001** |
|  | R^2^(Δ~Δ‘)↑ | -3.759±0.108 | -2.784±0.094 | -16.865±7.357 | -1.985±0.101 | **-1.416±0.014** |
|  | RMSE(Δ~Δ‘)↓ | 1.316±0.011 | 1.206±0.013 | 2.165±0.439 | 1.042±0.014 | **0.95±0.003** |

Note: Results are reported as the mean ± standard deviation over three random seeds. Bold indicates the best performance, and underlined values denote the second best. Metric directionality is indicated by arrows.

**Supplementary Table 2.** Ablation study on cell-line descriptions.

|  | cross-drug | | cross-cell line | | strict cross-cell line | |
| --- | --- | --- | --- | --- | --- | --- |
|  | PertDiff-no | PertDiff | PertDiff-no | PertDiff | PertDiff-no | PertDiff |
| Pearson(x~x’)↑ | 0.967±0.000 | **0.969±0.000** | 0.914±0.002 | **0.917±0.001** | 0.904±0.001 | **0.907±0.001** |
| Pearson(Δ~Δ‘)↑ | 0.610±0.001 | **0.629±0.001** | 0.324±0.003 | **0.337±0.002** | 0.328±0.000 | **0.334±0.001** |
| PP@100(Δ~Δ‘)↑ | 0.441±0.002 | **0.454±0.001** | 0.257±0.003 | **0.267±0.005** | **0.259±0.001** | 0.259±0.002 |
| NP@100(Δ~Δ‘)↑ | 0.453±0.001 | **0.464±0.001** | **0.240±0.002** | 0.234±0.002 | 0.238±0.001 | **0.243±0.001** |
| R^2^(Δ~Δ‘)↑ | 0.331±0.002 | **0.366±0.001** | -1.325±0.053 | **-1.323±0.047** | -1.482±0.009 | **-1.416±0.014** |
| RMSE(Δ~Δ‘)↓ | 0.529±0.001 | **0.516±0.000** | 0.890±0.009 | **0.881±0.006** | 0.964±0.002 | **0.95±0.003** |

Note: Results are reported as the mean ± standard deviation over three random seeds. Bold indicates the best performance, and underlined values denote the second best. Metric directionality is indicated by arrows.

**Supplementary Table 3.** Detailed Data Splits.

| Scenario | Task | Split unit | Training | Validation | Test | Notes |
| --- | --- | --- | --- | --- | --- | --- |
| Cross‑drug | Perturbation prediction | Drug | 60% | 20% | 20% | Following TranSiGen |
| Cross‑cell | Perturbation prediction | Cell | 150 | 7 | 7 | Following TranSiGen |
| Strict cross‑cell | Perturbation prediction | Cell | 10 | 2 | 3 | Following TranSiGen |
| Cross‑drug | Drug sensitivity | Drug | 60% | 20% | 20% | 7,148 drugs held out for inference |
| Cross‑cell | IC_50_ regression | Cell | 138 | 6 | 6 | 14 cell lines held out for inference |

Note: Perturbation prediction splits (based on 78,569 drug‑cell combinations, 164 cell lines, 8,316 drugs). Cross‑drug scenario: Split by drug. Training: 60% of drugs (4,989 drugs), validation: 20% (1,664 drugs), test: 20% (1,663 drugs). No drug overlap. Cross‑cell scenario: Split by cell line following TranSiGen. Training: 150 cell lines, validation: 7, test: 7. Strict cross‑cell scenario: Following TranSiGen. Training: 10 cell lines, validation: 2, test: 3. Drug response prediction splits: Sensitive drug classification (cross‑drug): 335 drugs split 60%(204 drugs)/20%(69 drugs)/20%(62 drugs). Remaining 7,148 drugs used for inference only. IC_50_ regression (cross‑cell): 14 cell lines with IC_50_ labels held out for inference. Remaining 150 cell lines split into training (138), validation (6), test (6). Fair comparison: All baseline models used the same splits in each scenario.

**Supplementary Table 4.** Predictive performance comparison of different models. Experiment is drug response prediction under cross drug scenario. That is, using a small-scale dataset for model training and testing, the test results are as follows.

| Drug split | TranSiGen | PertDiff-no | PertDiff |
| --- | --- | --- | --- |
| Pearson(x~x’)↑ | 0.951 | 0.953 | **0.955** |
| Pearson(Δ~Δ‘)↑ | 0.528 | 0.537 | **0.554** |
| PP@100(Δ~Δ‘)↑ | 0.37 | 0.377 | **0.385** |
| NP@100(Δ~Δ‘)↑ | 0.388 | 0.397 | **0.408** |
| R^2^(Δ~Δ‘)↑ | -0.194 | **0.179** | 0.148 |
| RMSE(Δ~Δ‘)↓ | 0.695 | 0.622 | **0.617** |

**Supplementary Table 5.** Number of drug-cell combinations in drug response prediction under cross drug scenario. The data is come from CTRP.

| Cell | Train drugs | Test drugs |
| --- | --- | --- |
| PC3 | 167 | 45 |
| MCF7 | 169 | 39 |
| A375 | 162 | 42 |
| HT29 | 145 | 34 |

**Supplementary Table 6.** Predictive performance comparison of different models. Experiment is drug response prediction under cross cell line scenario. That is, using a small-scale dataset for model training and testing, the test results are as follows.

| Cell split | TranSiGen | PertDiff-no | PertDiff |
| --- | --- | --- | --- |
| Pearson(x~x’)↑ | 0.893 | 0.905 | **0.918** |
| Pearson(Δ~Δ‘)↑ | 0.353 | 0.295 | **0.385** |
| PP@100(Δ~Δ‘)↑ | 0.258 | 0.243 | **0.283** |
| NP@100(Δ~Δ‘)↑ | 0.271 | 0.242 | **0.277** |
| R^2^(Δ~Δ‘)↑ | -1.123 | -0.898 | **-0.583** |
| RMSE(Δ~Δ‘)↓ | 1.051 | 1.017 | **0.921** |

**Supplementary Table 7.** Number of drug-cell combinations in drug response prediction under cross cell scenario. The data is come from GDSC2.

| Cell | Drugs | Lable | Cell | Drugs | Lable | Cell | Drugs | Lable |
| --- | --- | --- | --- | --- | --- | --- | --- | --- |
| 5637 | 103 | Train | J82 | 104 | Train | YAPC | 106 | Train |
| A204 | 104 | Train | MCF7 | 107 | Train | 22RV1 | 106 | Test |
| A375 | 113 | Train | MKN45 | 106 | Train | A549 | 106 | Test |
| AGS | 104 | Train | OVTOKO | 104 | Train | T47D | 107 | Test |
| BEN | 109 | Train | RCC10RGB | 104 | Train |  |  |  |
| Total sig = 1483 | | | | | | | | |

**Supplementary Table 8.** Principal component selection for E‑distance calculation.

| Cohort | Effective sample size (after resampling) | Number of PCs retained |
| --- | --- | --- |
| GSE32646 | 108 | 10 |
| GSE20194 | 224 | 20 |
| GSE25055 | 488 | 30 |

Note: Effective sample size = 2 × (2 × min(num(RD), num(pCR))), accounting for both control and predicted perturbed profiles.

**References**

DeepSeek. DeepSeek language model. In.; 2025.

Glorot, X. and Bengio, Y. Understanding the difficulty of training deep feedforward neural networks. In, *Proceedings of the thirteenth international conference on artificial intelligence and statistics*. JMLR Workshop and Conference Proceedings; 2010. p. 249–256.

Institute, B. Expanded CMap LINCS Resource 2020. In.; 2020.

Lamb, J.*, et al.* The Connectivity Map: using gene-expression signatures to connect small molecules, genes, and disease. *science* 2006;313(5795):1929–1935.

Li, P.*, et al.* An effective self-supervised framework for learning expressive molecular global representations to drug discovery. *Briefings in Bioinformatics* 2021;22(6):bbab109.

OpenAI. ChatGPT. In.; 2025.

Peidli, S.*, et al.* scPerturb: harmonized single-cell perturbation data. *Nature Methods* 2024;21(3):531–540.

Reimers, N. and Gurevych, I. Sentence-bert: Sentence embeddings using siamese bert-networks. *arXiv preprint arXiv:1908.10084* 2019.

Subramanian, A.*, et al.* A next generation connectivity map: L1000 platform and the first 1,000,000 profiles. *Cell* 2017;171(6):1437–1452. e1417.

Tong, X.*, et al.* Deep representation learning of chemical-induced transcriptional profile for phenotype-based drug discovery. *Nature Communications* 2024;15(1):5378.

Yang, W.*, et al.* Genomics of Drug Sensitivity in Cancer (GDSC): a resource for therapeutic biomarker discovery in cancer cells. *Nucleic acids research* 2012;41(D1):D955–D961.
